# Establishing the Role of Stereotactic Ablative Body Radiotherapy in Early-Stage Breast Cancer

**DOI:** 10.1155/2018/2734820

**Published:** 2018-02-01

**Authors:** Aisling Barry, Anthony Fyles

**Affiliations:** Department of Radiation Oncology, Princess Margaret Cancer Centre, University of Toronto, 610 University Avenue, Toronto, ON, Canada

## Abstract

Stereotactic ablative body radiotherapy (SABR) has a role as definitive therapy in many tumor sites; however, its role in the treatment of breast cancer is less well explored. Currently, SABR has been investigated in the neoadjuvant and adjuvant setting with a number of ongoing feasibility studies. However, its use comes with a number of radiobiological and technical challenges that require further evaluation. We have learned much from other extracranial disease sites such as lung, brain, and spine, where definitive treatment with SABR has shown encouraging outcomes. In women with breast cancer, SABR may eliminate the need for invasive surgery, reducing healthcare costs and hospital stays and providing an additional curative option for early-stage disease. This poses the following question: is there a role for SABR as a definitive therapy in breast cancer?

## 1. Introduction

In Canada, breast cancer is the third most common cancer after lung and colorectal cancer and is the most common cancer diagnosis in females. As our population continues to live longer, we will see an increase in those diagnosed with breast cancer over the age of 70. According to the 2017 Canadian Cancer Statistics [1], one-third of new breast cancer diagnoses are 70 years or older. The role of radiotherapy in the management of breast cancer is well established in the adjuvant setting [2]. However, it is increasingly recognized that short course partial breast radiation may be an option particularly in older low-risk women [3] and some may not require radiation at all [4]. Therefore, finding additional options for treatment reduction and improved efficiency within cancer care is important for both patients and our healthcare system.

There is little clinical data supporting the use of definitive conventionally fractionated radiotherapy [5, 6] and more specifically stereotactic ablative body radiotherapy (SABR) in breast cancer. Many sites, including lung, brain, spine, and prostate, have adopted SABR as a definitive treatment option in early-stage disease. Stereotactic ablative body radiotherapy has many advantages by delivering high doses of radiation over a short period and saving treatment days, overall time on the treatment machine, and overall cost. Patients, if treated definitively, may never require surgical intervention, eliminating the need for anesthetic and inpatient hospital stays, with a potential further reduction in health and hospital expenditure.

In this review, we discuss the current radiotherapy standards, where SABR has been studied and its potential role and challenges as a definitive treatment in early-stage breast cancer.

### 1.1. Current Standards of Treatment in Early-Stage Breast Cancer

Adjuvant radiotherapy following lumpectomy and sentinel lymph node biopsy is standard of care in early-stage breast cancer patients [2]. In recent times, breast cancer radiotherapy has evolved from conventional fractionated radiotherapy (1.8 Gy–2 Gy per fraction) to hypofractionated radiotherapy (>2 Gy per fraction), taking advantage of the perceived low alpha/beta ratio of breast cancer cells [7, 8].

Hypofractionation has been investigated in a number of prospective randomized studies [3, 8] and has been integrated worldwide into standard treatment protocols in early-stage breast cancer. These studies have demonstrated equivalent local control outcomes with minimal differences in side effects compared to standard fractionation schedules.

An additional option in early-stage breast cancer is the use of accelerated partial breast irradiation (APBI). As most local recurrences after breast conserving therapy occur in or close to the tumor bed [9], APBI aims to deliver high doses of radiation around the tumor bed to a limited volume of breast tissue over a short period. Options for APBI treatment delivery include conformal external beam radiation, interstitial brachytherapy, intracavitary brachytherapy, and intraoperative radiation. Two randomized trials comparing whole breast to partial breast irradiation [10, 11] showed partial breast irradiation to be a safe and effective treatment option with low recurrence rates in a well-selected cohort of early-stage breast cancer patients. The RAPID [12] study compared twice daily fractionated radiotherapy to standard whole breast radiotherapy with unfavorable cosmetic and late radiotherapy toxicity outcomes. Additionally, a recent Cochrane review [13] of patients receiving APBI versus whole breast irradiation found no difference in loco regional recurrence-free survival, cause-specific survival, distant metastasis-free survival, or mastectomy rates. Late subcutaneous fibrosis and telangiectasia were worse in the APBI cohort; however, longer follow-up results are still needed. The American Society for Radiation Oncology recently updated the APBI consensus statement to include patients above the age of 50 years, T1 disease, and DCIS less than 2.5 cm, as well as ER positive [14].

### 1.2. Radiotherapy Alone as Definitive Treatment in Breast Cancer

Radiotherapy's role as a definitive ablative therapy (i.e., without surgery) is less well considered and to the best of our knowledge there are no prospective comparative studies in the literature of the current standard (surgery +/− radiotherapy +/− chemotherapy/hormonal therapy) versus definitive radiotherapy. Age and medical comorbidities often deem patients inoperable and, in general, these patients are then treated with palliative intent using hormonal therapy alone or palliative radiotherapy if applicable.

A collaborative study between the Gustave-Roussy Institute and the Princess Margaret Hospital [5] reviewed the use of radiotherapy alone as definitive breast cancer treatment. Patients who had inoperable disease or who were unable to undergo general anesthesia received definitive hypofractionated radiotherapy (40 Gy in 16 fractions, 45 Gy in 20 fractions, or 45 Gy in 18 fractions). The retrospective study demonstrated tumor dose as being a highly significant factor in local disease control (Table 1) and on a subsequent review of the patient cohort 10 years later, the group described the incidence of disabling complications as low and as expected related to total dose.

Van Limbergen et al. [6] also performed a retrospective analysis of 221 patients with Tis-T3 N0-1 breast cancer treated with radiotherapy alone. The risk of local recurrence was significantly associated with the size of tumor, age, radiation dose (Table 1), and length of split course intervals. They concluded that doses needed to provide local control similar to a combination of surgery and radiation are 10 Gy higher for T1 tumors and 35 Gy higher for T2 tumors.

Unfortunately, higher doses may result in worsened cosmetic outcomes, as a separate adjuvant breast dose escalation paper by Van Limbergen et al. [15] reported. The authors reviewed 161 patients; those that received higher than 75 Gy in 37 fractions lead to very poor results in more than 30% of patients and only 15% of patients who received more than 80 Gy resulted in good cosmetic results. Additionally, in a paper by Calle et al. [16], who treated 394 patients with doses of up to 8500 rad (85 Gy) alone (no surgery), only 38% of patients reported a good-to-excellent 5-year cosmetic outcome. However, it is important to remember that both of these papers are more than 25 years old, using older radiotherapy techniques and larger treatment fields.

### 1.3. Clinical Application of SABR in Breast Cancer

Key advancements have been made in the delivery of hypofractionated radiotherapy, including advances in the delivery of more homogenous doses of radiation and radiobiological understanding of more definite dose equivalent estimation [17]. Stereotactic ablative body radiotherapy in breast cancer is appealing and has been shown to be a safe and effective definitive treatment option in many tumor sites, including lung, brain, and liver [18, 19]. Timmerman et al. [19] published one of the earlier extracranial SABR trials on its use in early-stage lung cancer, demonstrating excellent local control rates and side effect outcomes in patients deemed medically inoperable.

To date, SABR has been investigated in the setting of neoadjuvant and adjuvant therapy in breast cancer treatment.

#### 1.3.1. Neoadjuvant SABR

Bondiau et al. [20] conducted a phase 1 study involving 25 patients to determine the maximum tolerable dose of SABR concomitant with neoadjuvant chemotherapy before surgery. One-third of patients had a pathological complete response, with the highest rate at dose level of 25.5 Gy in 3 consecutive fractions. However, the maximum tolerated dose was not reached as the group found that early SABR related toxicities were rare.

There are a number of ongoing phase I/II studies exploring the use of SABR in the neoadjuvant setting (Table 2). Currently, in Canada, the Juravinski Cancer Centre is conducting a phase I feasibility study of the role of SBRT for the treatment of early-stage breast cancer called ARTEMIS [21], which will deliver 40 Gy in 5 fractions every other day to the gross tumor, followed by breast conserving surgery. The primary outcome measure is feasibility and successful delivery of SABR.

Similarly, in Netherlands, a multicenter single-arm prospective study called the ABLATIVE study [22] is recruiting patients to undergo MR-guided single-dose APBI with an integrated boost, followed by breast conserving surgery at 6 months. The primary study point aim is to assess pathological complete response and secondarily to review radiological response and toxicity.

#### 1.3.2. Adjuvant SABR

Stereotactic accelerated partial breast irradiation for early-stage breast cancer in the adjuvant setting has been studied in a small prospective trial [26] which looked at ten patients who received CyberKnife therapy. Each received a total dose of 30 Gy in five consecutive fractions. The group concluded that early findings show CyberKnife as a feasible, well-tolerated, and reliable platform for APBI.

### 1.4. Radiobiology, Toxicity, and Technical Challenges in Using SABR

#### 1.4.1. Radiobiology and Toxicity

One of the many advantages of SABR is the ability to deliver large doses of radiation to the tumor while sparing surrounding normal tissue. Similar to prostate cancer, it has been hypothesized that breast tissue is sensitive to fraction size; that is, the larger the dose per fraction the higher the tumor cell kill; hence the application of hypofractionation in breast cancer is aiming to take advantage of this phenomenon [27].

Unfortunately, one of the drawbacks to delivering larger radiation doses is the increased risk of late normal tissue toxicity. Cosmetic breast outcomes from the hypofractionated trials have to date been acceptable, bearing in mind the fact that they refer to whole breast irradiation [3, 8], with poorer outcomes associated with large excision volume and delayed wound healing postoperatively. Also, the RAPID study trialists [12] reported significantly worse nurse, patient, and physician reported cosmetic outcomes compared to the standard fractionation group. So far, in the small SABR studies described in this review, cosmetic and breast outcomes have been acceptable. However, with these early results, where cosmetic outcome was not a primary endpoint, it is important to remember that potential adverse cosmetic outcomes may not be seen for many more years. Regardless, there is still need for clarity with respect to optimal dose fractionation schedules to avoid significant late effects.

#### 1.4.2. Potential Technical Challenges

Safe delivery of large doses of radiation to any tumor site requires several rigorous quality assurance steps. The challenge, as with any radiotherapy plan, is location of disease and reproducibility. This is almost certainly even more relevant for patients receiving very high doses of radiation. Therefore, the use of breast molds and/or rigid immobilization may be required for patients being treated in the supine position.

Like lung and liver SABR, technical considerations such as 4DCT (4-dimensional computed tomography) and motion management using deep inspiration breath hold or active breath control may be useful in reviewing and managing the patients' breathing cycle, thus reducing or eliminating tumor motion and in turn reducing planning margins. These methods are currently widely used to reduce heart dose in patients receiving left-sided breast radiotherapy. Of note, Bondiau et al. [20] and Obayomi-Davies et al. [26] both used CyberKnife System (Accuracy Inc., Sunnyvale, CA) as primary delivery method by means of real-time respiratory tracking of implanted fiducial markers.

In addition to contrast-enhanced CT simulation, MRI as a simulation tool would be useful in providing superior soft tissue contrast and offering improved visualization of the breast tumor, with potential scope in the future for treatment delivery via an MR-guided linear accelerator. One of the challenges recognized is that breast cancer patients receiving a diagnostic MRI are in the prone position, while for reproducibility and consistency in radiation treatment, patients are treated in a supine position. A wide bore MRI scanner may then be required in order to acquire supine images in the radiotherapy position. Den Hartogh et al. [28] designed an MRI protocol incorporating a wide bore scanner and demonstrated high target volume delineation consistency among observers. Schmitz et al. [29] compared preoperative MRI-derived gross tumor volume (GTV) to histopathological measurements of the tumor in addition to any subclinical disease. There was good correlation between size of the visible MRI tumor and that measured at pathology (Pearson's correlation: 0.76). Only 10% of patients had invasive disease beyond 10 mm of the MRI-GTV, with the likelihood of subclinical disease greater in tumors with extensive intraductal components. In addition, preoperative delineation is shown to have less interobserver variability [30] compared with postoperative volume delineation and the expansion to a clinical target volume used to account for microscopic disease is not often employed during SABR treatment but may be considered in patients with potential adverse features, thus potentially reducing the volume of normal tissue in the treatment field. Fiducial markers were used in the neoadjuvant and adjuvant SABR studies [20, 26], which can help with tumor identification and volume delineation, in addition to tracking and locating treated disease sites on follow-up surveillance. An internal target volume is used to account for organ motion; this can be estimated by assessing the motion between the inhale and exhale phases on the 4DCT.

Defining organs at risk and dose constraints to normal tissues continues to be a challenge in the world of SABR. It is generally not recommended to extrapolate from dose constraints associated with conventional fractionation. Constraints may be extrapolated from prior lung and liver SABR trials, but this in turn is somewhat restricted.

#### 1.4.3. Posttreatment Follow-Up

A challenge when delivering any type of definitive treatment is follow-up surveillance. We have learned from our lung colleagues that imaging changes can be confusing and often related to inflammation or fibrosis [31]. Definitive breast radiotherapy treatment we suspect will be no different and defining disease response and disease progression on what modality and frequency of follow-up imaging will require extensive review. Considerations to using CT, MRI, and functional imaging techniques such as PET-CT may be required to aid in this differentiation.

## 2. Conclusion

As we continue to strive to deliver the best of patient care with more patient convenience and less healthcare costs, it may only be a matter of time before SABR is integrated into the breast cancer treatment paradigm. With such rapid advancements in radiotherapy technology, it is imperative that well-constructed multi-institutional collaborative feasibility and randomized trials are developed. These should aim to explore the technique of SABR further, especially in early-stage elderly breast cancer patients and, in addition, to address the many radiobiological, technical, and toxicity issues that may arise through its use to deliver safe, quality-assured, evidence-based radiotherapy.

## Figures and Tables

**Table 1:** Local recurrence risk and tumor dose.

Tumor dose (Gray)	Local recurrence risk	
	Arriagada et al. [5]	Van Limbergen et al. [6]
	% (number of patients)	% (number of patients)
≤50	77% (99/128)	75% (3/4)
51–60	68.7% (55/81)	21% (4/19)
61–70	54.8% (17/31)	16.6% (2/12)
71–80	31.7% (33/104)	24.4% (12/49)
>80	24% (13/54)	12.5% (17/136)

**Table 2:** Ongoing phase I/II studies exploring the use of SABR in the neoadjuvant setting.

Name of study	Estimated number to be enrolled	Inclusion	Primary endpoints	SABR dose
Feasibility Study of Stereotactic Body Radiotherapy for Early Breast Cancer (ARTEMIS) [21]	32	Women ≥ 70 yr with preoperative early-stage breast cancer, followed by lumpectomy at 8–12 weeks after SABR	Treatment feasibility	40 Gy in 5 fractions every other day
Single Dose Ablative Radiation Treatment for Early-Stage Breast Cancer (ABLATIVE) [22]	25	Core biopsy positive nonlobular carcinoma, with negative sentinel lymph node biopsy followed by lumpectomy 6 months after SABR	Pathological complete response	20 Gy in 1 fraction
Preoperative Single-Fraction Radiotherapy in Early Stage Breast Cancer [23]	100	Women ≥ 50 yr, biopsy proven, CT1N0, ER +ve, invasive ductal, or DCIS, followed by lumpectomy 8–12 weeks after SABR	Rate of pathological response at time of surgery	21 Gy in 1 fraction
Stereotactic Image-Guided Neoadjuvant Ablative Radiation Then Lumpectomy (SIGNAL) [24]	120	Postmenopausal women ≥ 55 yr, ≤3 cm, ER +ve, clinically node negative, invasive ductal carcinoma, followed by lumpectomy 6–8 weeks after SABR	Toxicity resulting from radiation	21 Gy in 1 fraction
Preoperative Stereotactic Ablative Body Radiotherapy (SABR) for Early-Stage Breast Cancer [25]	40	Women ≥ 50 yr, invasive adenocarcinoma, ≤2 cm, followed by lumpectomy 6 weeks after SABR	Rate of pathological complete response	3 fractions
